# Effect of distal access catheter tip position on angiographic and clinical outcomes following thrombectomy using the combined stent-retriever and aspiration approach

**DOI:** 10.1371/journal.pone.0252641

**Published:** 2021-06-10

**Authors:** Sang Hun Baek, Sanghyeon Kim, Myongjin Kang, Jae-Hyung Choi, Hee Jin Kwon, Dong Won Kim

**Affiliations:** 1 Department of Radiology, Busan Regional Cardio-Cerebrovascular Disease Center, Dong-A University Hospital, Busan, Republic of Korea; 2 Department of Neurosurgery, Busan Regional Cardio-Cerebrovascular Disease Center, Dong-A University Hospital, Busan, Republic of Korea; National University of Ireland - Galway, IRELAND

## Abstract

**Purpose:**

Mechanical thrombectomy using the stent-retriever in conjunction with the distal access catheter may improve the rates of successful revascularization and clinical outcomes in patients with acute stroke. We aimed to compare two different thrombectomy techniques, according to the position of the distal access catheter tip in the combined stent-retriever and aspiration approach.

**Methods:**

In this retrospective study, patients with middle cerebral artery occlusion treated with the combined technique were divided into two groups based on the tip position of the distal access catheter: distal group (catheter placed adjacent to the thrombus) and proximal group (catheter placed in the cavernous segment of the internal carotid artery below the ophthalmic artery). Baseline characteristics, angiographic results, and clinical outcomes were compared.

**Results:**

Eighty-three patients (distal group, n = 45; proximal group, n = 38) were included. Higher complete reperfusion was observed in the distal group (unweighted analysis: 66.7% vs. 42.1%, p = 0.025; weighted analysis: 74.0% vs. 28.8%; p = 0.002). In the multivariate analysis, the distal tip position was independently associated with complete reperfusion (unweighted analysis: aOR, 4.10; 95% CI, 1.40–11.98; p = 0.01; weighted analysis: aOR, 5.20; 95% CI, 1.72–15.78; p = 0.004). The distal group also showed more favorable clinical outcomes and early neurological improvement (unweighted analysis: 62.2% vs. 55.3%; p = 0.521, 60% vs. 50%; p = 0.361, respectively; weighted analysis: 62.7% vs. 61.1%; p = 0.877, 66% vs. 45.7%; p = 0.062, respectively). However, more arterial dissections were observed in the distal group (8.9%, n = 4 vs. 2.6%, n = 1; p = 0.36). In the distal group, one patient with vascular injury died due to complications. No cases of emboli in new territory were observed.

**Conclusions:**

Distal tip position of the distal access catheter has a significant impact on reperfusion in patients with acute ischemic stroke. However, there was also a higher rate of vascular injury as the catheter was advanced further. If advancement to the target lesion is too difficult, placing it in the cavernous internal carotid artery may be a viable method without complications.

## Introduction

With five large randomized clinical trials in 2015 demonstrating the superiority of endovascular treatment over medical treatment in acute ischemic stroke caused by large vessel occlusion [1–5], neurointerventionalists are striving to refine their approaches to increase revascularization rates using new devices and techniques. The problem frequently encountered with stent-retriever thrombectomy, the standard procedure for mechanical thrombectomy, is distal thromboembolism during thrombectomy (with 7% cases reported in the TREVO 2 trial) [6]. Stent-retriever thrombectomy in conjunction with a distal access catheter (DAC), when compared with conventional thrombectomy (guide catheter with only stent-retriever), led to improved rates of revascularization and clinical outcomes [7]. Localized aspiration at the site of the thrombus may promote entrapment of the thrombus within the stent. Additionally, flow-control in the affected vascular territory may reduce the incidence of thrombus fragmentation and distal embolization. The aspiration force and effect of the DAC in the combined stent-retriever and aspiration technique are expected to produce optimal results when the distance between the distal tip position of the DAC and the thrombus is short. However, advancing the DAC to the target occlusion might be difficult in cases with tortuous vascular anatomy, where there is a risk of damage to the vasculature. Moreover, the true impact of the DAC tip position in the combined technique is not well known. Thus, the purpose of our study was to compare the actual effect of DAC tip positions on angiographic and clinical outcomes following thrombectomy using the combined stent-retriever and aspiration technique.

## Materials and methods

### Patients

We retrospectively reviewed consecutive patients with acute stroke who underwent mechanical thrombectomy at the Dong-A University Hospital, a tertiary stroke center, between November 2016 and November 2019. Patient data were obtained from a prospectively maintained stroke registry. Our research team accessed the records on January 2020 to obtain the patient data.

The inclusion criteria for this study were as follows: 1) acute ischemic stroke within 6 hours of symptom onset; 2) intracranial large-vessel occlusion involving the middle cerebral artery (MCA) (M1 or proximal M2); 3) preprocedural diffusion-weighted imaging (DWI); and 4) endovascular procedure using an intermediate catheter (6F Catalyst DAC, Stryker Neurovascular, Fremont, CA, USA) and a stent-retriever (Trevo, Stryker Neurovascular, Fremont, CA, USA), with simultaneous use of a balloon guide catheter (BGC). Patients with ipsilateral stenosis, tandem occlusions, or dissection as the etiology of stroke were excluded.

Based on the location of the DAC tip within the vessel, the cases were divided into two groups: distal DAC group (the DAC tip was positioned adjacent, as close as possible, to the proximal surface of the thrombus in the MCA), and proximal DAC group (the DAC tip was placed in the cavernous segment of the ICA below the ophthalmic artery). This study was approved by the Institutional Review Board and the requirement of informed consent was waived, based on the retrospective nature of the study (DK-IIT-2019-14).

### Data collection and outcome measurement

Data on baseline characteristics, angiographic results, and clinical outcomes were obtained from the medical records. Baseline characteristics included sex; age; intravenous tissue plasminogen activator (tPA) therapy; history of hypertension, diabetes mellitus, smoking, hyperlipidemia, coronary artery disease, atrial fibrillation, and ischemic stroke or transient ischemic attack; initial National Institute of Health Stroke Scale (NIHSS) score; infarct volume on DWI; and internal carotid artery (ICA) tortuosity. Infarct volume was determined by measuring the hypointense regions on the apparent diffusion coefficient map (thresholds: 600 × 10^−6^ mm^2^/s) [8]. Based on the geometry of the anterior and posterior genus in the ICA, the tortuosity of the cavernous ICA was classified into three categories: mild, moderate, and severe [9].

Angiographic parameters included presence of collateral flow from the anterior communicating (ACom) artery or posterior communicating (PCom) artery, total number of thrombectomy attempts, procedural time (groin puncture to recanalization time), embolization in new territory, modified Thrombolysis in Cerebral Infarction (mTICI) score, and procedure-related complications. The collateral flow from the ACom or PCom artery was considered to be present if the contralateral A1 segment or ipsilateral PCom artery, except the fetal variant, was visible during angiography. If there was severe stenosis or occlusion of contralateral ICA or posterior circulation, we assumed that there was no collateral flow from either the ACom or PCom artery. Emboli in new territory was defined as emboli observed on post-thrombectomy angiography within the previously unaffected territories [10]. Successful reperfusion was defined as mTICI scores of 2b or 3. Complete reperfusion was defined as an mTICI score = 3 [11]. Clinical outcomes were assessed based on early neurological improvement, functional outcome, modified Rankin Scale (mRS) [12], mortality, and occurrence of symptomatic intracerebral hemorrhage. Early neurological improvement was defined as an NIHSS score improvement of ≥8 within 24 hours, or an NIHSS score of 0–1 at 24 hours [13]. A good clinical outcome was defined as 3-month mRS scores of 0, 1, and 2. Mortality was defined as death occurring within 90 days. Symptomatic intracerebral hemorrhage was defined as any intracranial hemorrhage associated with a worsening of NIHSS score by ≥4 within 24 hours [14].

The angiographic assessment was performed by a single neurointerventionalist who was not involved in the mechanical thrombectomy. A certified stoke neurologist, who was blinded to the neurointerventional approach used in the study, assessed the clinical outcomes.

### Endovascular procedure

**Fig 1:**
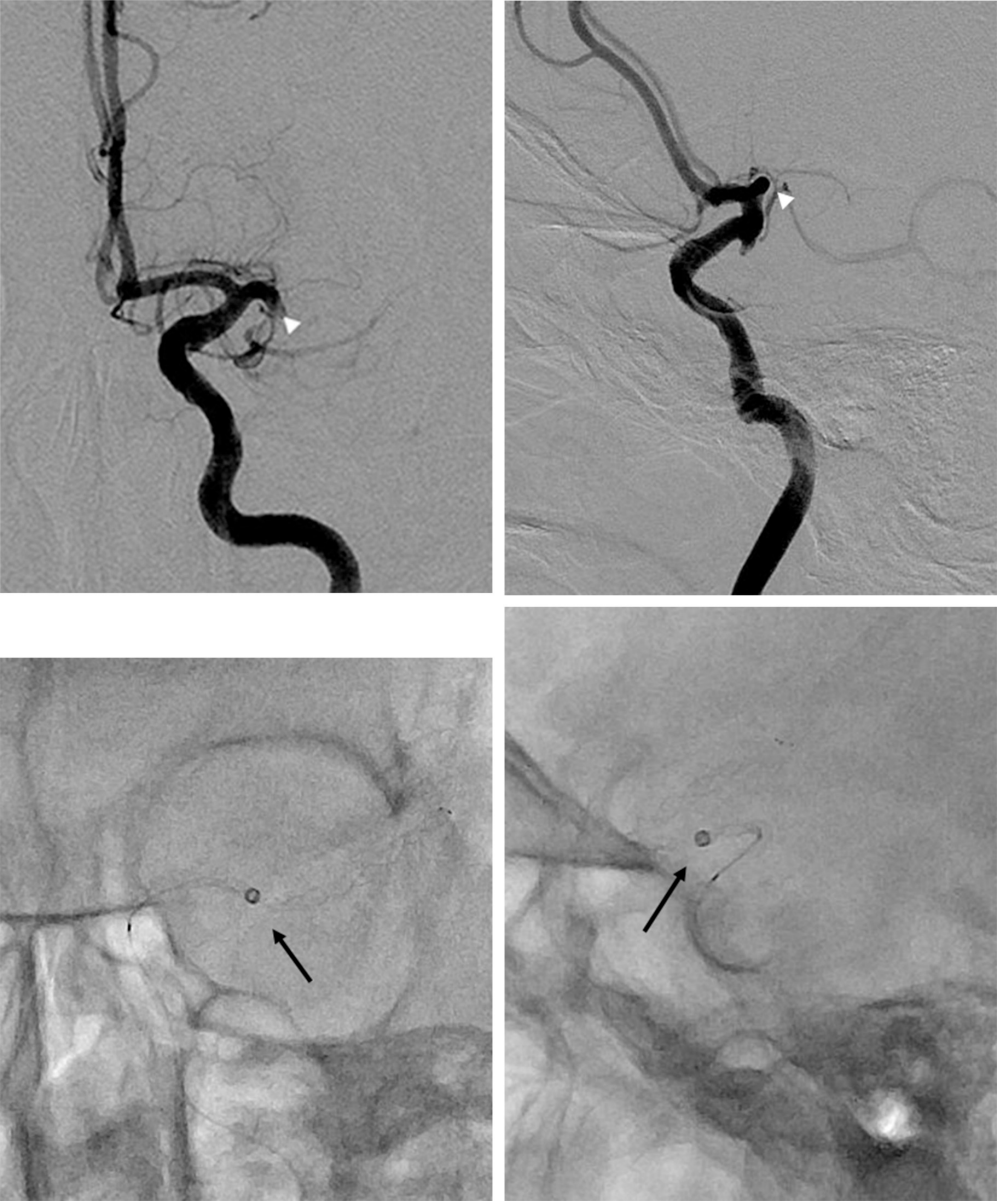
Distal access catheter (DAC) tip position during combined stent-retriever and aspiration technique in the distal DAC group. (A, B) Left anteroposterior (A) and lateral (B) internal carotid artery angiogram images show occlusion of the left middle cerebral artery (arrowheads). (C, D) The DAC was advanced as close to the proximal end of the thrombus as possible in the middle cerebral artery (arrows).

**Fig 2:**
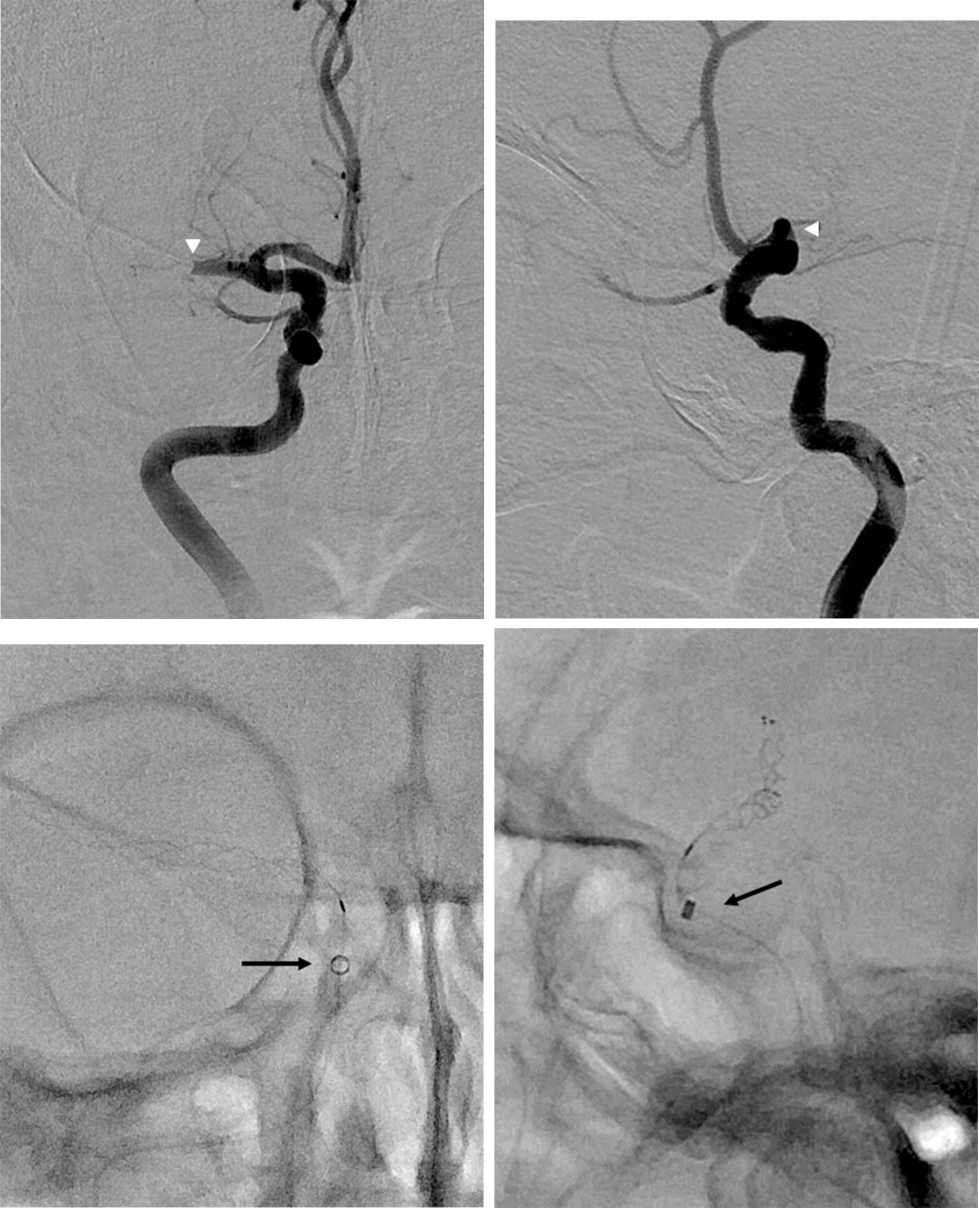
Distal access catheter (DAC) tip position during combined stent-retriever and aspiration technique in the proximal DAC group. (A, B) Right anteroposterior (A) and lateral (B) internal carotid artery angiogram images show occlusion of the right middle cerebral artery (arrowheads). (C, D) The DAC was placed in the cavernous ICA below the ophthalmic artery origin (arrows).

All procedures were performed via transfemoral puncture. A BGC (8F Merci, Stryker Neurovascular or 8F Optimo, Tokai Medical) was introduced into the cervical segment of the ICA on the affected side. The DAC (6F Catalyst DAC; Stryker Neurovascular, Fremont, CA, USA) was subsequently advanced into the distal ICA, using a coaxial technique with a microcatheter over a microwire. Next, the microcatheter was placed distal to the thrombus, and the stent-retriever (Trevo, Stryker Neurovascular, Fremont, CA, USA) was deployed through the thrombus; 3−4 minutes were allowed for clot integration. Subsequently, the microcatheter was slowly retracted (bare wire technique) to maximize the DAC’s cross-sectional area for aspiration [14]. The DAC was advanced, as close to the proximal end of the thrombus as possible, in the MCA (Fig 1) or placed in the cavernous segment of the ICA, below the ophthalmic artery origin (Fig 2). The location of the DAC tip within the vessel depended on the operator’s preference. The two neurointerventionalists performed the thrombectomy using different methods of the combined technique. One operator performed thrombectomy with the catheter placed in the cavernous ICA. The other performed thrombectomy with the catheter placed adjacent to the thrombus.

Advancing the DAC through the tortuous vessel segments and the ophthalmic artery origin can be difficult. In such cases, we used the microcatheter with a bulbous distal end (AXS *Offset*™ delivery assist catheter, *Stryker*, Fremont, CA, USA), a special catheter that reduces the space between the guidewire and DAC, or a steam-shaped distal tip of the DAC. The balloon was inflated to create a flow-arrest just before the retraction of the stent-retriever; the stent was slowly retracted into the DAC with continuous negative suction. Aspiration was performed using an electrical pump (Pump MAX, Penumbra). In case of blood flow blockade in the DAC, the stent-retriever-DAC complex was withdrawn as a unit into the BGC. Additional aspiration was performed using the BGC, prior to angiography.

### Statistical analysis

All statistical analyses were performed using the SPSS (version 25.0; SPSS Inc., Chicago, IL, USA). The various baseline characteristics, reperfusion rates, and outcomes were compared between the two groups. The categorical variables were compared using the Fisher’s exact test, chi-square tests, Student’s t-test or Mann−Whitney U test. Normality of distribution was checked using the Kolmogorov−Smirnov test. Successful reperfusion and complete reperfusion were analyzed for all cases of each group using multivariate logistic regression analysis. Variables with *p*-value <0.2 in the univariate analysis were selected for the logistic regression analysis [15].

To reduce selection bias, an inverse-probability-of-treatment-weighted (IPTW) approach based on the propensity score was used. The propensity score was constructed using a multiple logistic regression model, and a stabilized average treatment effect weight was used. The covariates for the adjustment strategies included sex; age; intravenous tPA; history of hypertension, diabetes mellitus, smoking, hyperlipidemia, previous coronary artery disease, atrial fibrillation, previous ischemic stroke or transient ischemic attack; initial NIHSS score; ischemic lesion volume; ICA tortuosity; collateral flows; number of thrombectomy attempts; and procedure duration.

The Hosmer−Lemeshow test was used to determine the goodness-of-fit for logistic regression models. A *p*-value of <0.05 was considered statistically significant. Results of the logistic regression analysis were reported as an adjusted OR (aOR) with 95% confidence interval (CI).

## Results

During the study period, a total of 210 patients with acute stroke underwent mechanical thrombectomy using the combined stent retriever-DAC technique. Among them, 101 patients had only MCA occlusion. The patients with different-profile stent-retriever or DAC (n = 10), underlying stenosis (n = 7), and dissection as etiology of stroke (n = 1) were excluded. Finally, 83 patients who met the criteria were enrolled. The DAC tip was located in the MCA in 45 patients (distal DAC group, 54.2%) and in the cavernous segment of the ICA in 38 patients (proximal DAC group, 45.8%). There was no switch to other techniques during the thrombectomy in all cases.

There were no significant differences between the groups in baseline characteristics, including sex; age; intravenous tPA therapy; history of hypertension, diabetes mellitus, smoking, hyperlipidemia, coronary artery disease, atrial fibrillation, ischemic stroke or transient ischemic attack; initial NIHSS score; ischemic lesion volume on DWI; or ICA tortuosity between the two groups in both the unweighted and IPTW analysis (Table 1).

**Table 1:** Comparison of the baseline characteristics according to the DAC tip position.

	Unweighted analysis			Inverse-probability-of-treatment-weighted analysis			
	Distal DAC group (n = 45)	Proximal DAC group (n = 38)	P value	Distal DAC group (n = 45)	Proximal DAC group (n = 38)	P value
Sex (male)	26 (57.8%)	24 (63.2%)	0.618	31 (60.8%)	24 (66.7%)	0.575
Age	70.93 ± 11.3	70.87 ± 10.0	0.978	71.41 ± 11.5	69.61 ± 10.1	0.454
Intravenous tPA	34 (75.6%)	26 (68.4%)	0.469	37 (74.0%)	23 (65.7%)	0.409
Hypertension	24 (53.3%)	27 (71.1%)	0.098	31 (60.8%)	22 (62.9%)	0.846
Diabetes mellitus	10 (22.2%)	15 (39.5%)	0.088	12 (50.0%)	12 (50.0%)	0.300
Smoking	20 (44.4%)	18 (47.4%)	0.790	22 (43.1%)	19 (52.8%)	0.375
Hyperlipidemia	10 (22.2%)	14 (36.8%)	0.143	15 (30.0%)	10 (28.6%)	0.887
Previous coronary artery disease	8 (17.8%)	3 (7.9%)	0.214	8 (15.7%)	3 (8.6%)	0.513
Atrial fibrillation	25 (55.6%)	21 (55.3%)	0.979	30 (60.0%)	19 (54.3%)	0.600
Previous ischemic stroke or TIA	8 (17.8%)	7 (18.4%)	0.940	8 (16.0%)	5 (14.3%)	0.829
Initial NIHSS score	13.82 ± 4.6	13.50 ± 4.7	0.755	14.14 ± 4.8	13.23 ± 4.6	0.380
Ischemic lesion volume on DWI	28.22 ± 26.4	29.68 ± 27.4	0.806	26.26 ± 24.9	31.34 ± 27.8	0.378
ICA tortuosity			0.657			0.729
Mild	23 (51.1%)	16 (42.1%)		22 (44.0%)	18 (50.0%)	
Moderate	18 (40.0%)	19 (50.0%)		24 (48.0%)	15 (41.7%)	
Severe	4 (8.9%)	3 (7.9%)		4 (8.0%)	3 (8.3%)	
Collaterals from Acom or Pcom artery	15 (33.3%)	12 (31.6%)	0.865	17 (33.3%)	12 (33.3%)	1.000

Data are expressed as mean ± standard deviation or numbers of patients (%).

DAC: distal access catheter; tPA: tissue plasminogen activator; TIA: transient ischemic attack; NIHSS: National Institute of Health Stroke Scale; DWI: diffusion weighted image; ICA: internal carotid artery; ACom, anterior communicating; PCom, posterior communicating.

With regard to successful reperfusion, no significant difference was observed between the distal and proximal DAC groups (unweighted analysis: 86.7% vs. 81.6%; p = 0.525; weighted analysis: 90.0% vs. 77.8%; p = 0.119). However, complete revascularization was achieved more often in the distal DAC group than in the proximal DAC group (unweighted analysis: 66.7% vs. 42.1%, p = 0.025; weighted analysis: 74.0% vs. 28.8%; p = 0.002). Although the distal DAC group had a more favorable outcome compared with the proximal DAC group, no significant differences were observed in the clinical outcomes and early neurological improvement between the two groups (unweighted analysis: 62.2% vs. 55.3%; p = 0.521, 60% vs. 50%; p = 0.361, respectively; weighted analysis: 62.7% vs. 61.1%; p = 0.877, 66% vs. 45.7%; p = 0.062, respectively). The angiographic and clinical outcomes are summarized in Table 2.

**Table 2:** Comparison of the outcomes between the distal and proximal DAC groups.

	Unweighted analysis			Inverse-probability-of-treatment-weighted analysis		
	Distal DAC group (n = 45)	Proximal DAC group (n = 38)	P value	Distal DAC group (n = 45)	Proximal DAC group (n = 38)	P value
Number of passes	1.64 ± 0.7	1.50 ± 0.8	0.406	1.56 ± 0.86	1.52 ± 0.73	0.840
Puncture-to-recanalization time (min)	64.887 ± 12.53	64.05 ± 10.81	0.755	62.63 ± 11.97	65.00 ± 10.57	0.347
Successful reperfusion (modified TICI≥2b)	39 (86.7%)	31 (81.6%)	0.525	45 (90.0%)	28 (77.8%)	0.119
Complete reperfusion (modified TICI = 3)	30 (66.7%)	16 (42.1%)	0.025	37 (74.0%)	15 (28.8%)	0.002
Favorable clinical outcome (mRS≤2 at 3 month)	28 (62.2%)	21 (55.3%)	0.521	32 (62.7%)	22 (61.1%)	0.877
Early neurological improvement	27 (60.0%)	19 (50.0%)	0.361	33 (66.0%)	16 (45.7%)	0.062
Arterial dissection	4 (8.9%)	1 (2.6%)	0.369	3 (75.0%)	1 (2.8%)	0.637
Symptomatic ICH	3 (6.7%)	0 (0%)	0.246	5 (9.8%)	0 (0%)	0.074
Emboli to new territory	0 (0%)	0 (0%)	1.000	0 (0%)	0 (0%)	1.000
Mortality	3 (6.7%)	2 (5.3%)	1.000	3 (6.0%)	2 (5.7%)	1.000

Data are expressed as mean ± standard deviation or numbers of patients (%).

DAC: distal access catheter; TICI: Thrombolysis in cerebral infarction; mRS: modified Rankin Scale; ICH: intracerebral hemorrhage.

**Fig 3:**
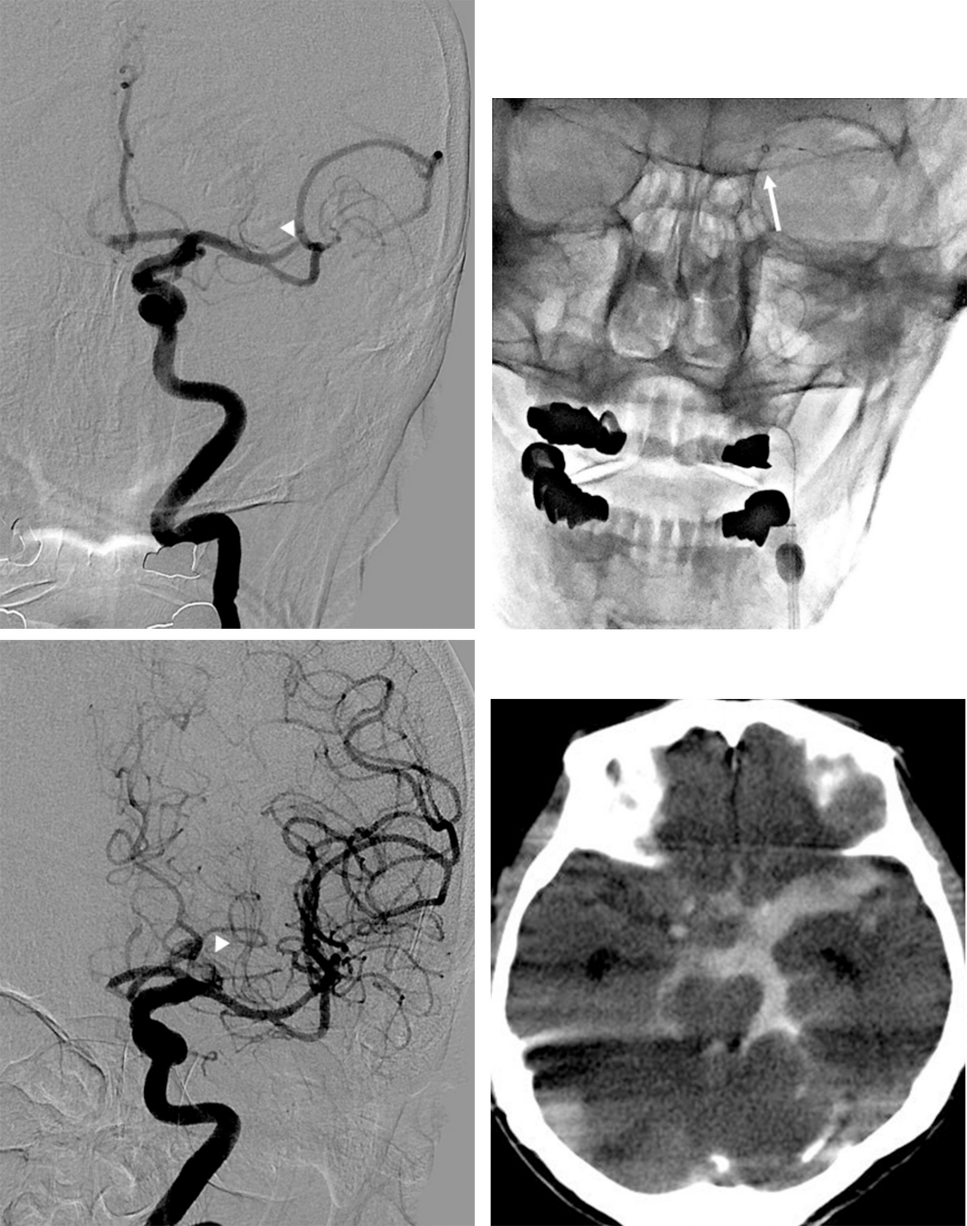
An 87-year-old man, who presented with acute stroke and an initial NIHSS score of 18. (A) Left anteroposterior internal carotid artery angiogram shows occlusion of the proximal superior trunk of the middle cerebral artery (arrowhead). (B) The advancement of the distal access catheter (DAC) in the middle cerebral artery was very difficult. The DAC was advanced in the proximal middle cerebral artery (arrow). (C) Post-thrombectomy anteroposterior internal carotid artery angiogram shows complete reperfusion of the middle cerebral artery; however, there is contrast extravasation (arrowhead) in the left proximal middle cerebral artery. (D) Postoperative axial CT shows subarachnoid hemorrhage.

Among the procedure-related complications, arterial dissection was more frequently observed in the distal DAC group; however, the results lacked statistical significance (8.9%, n = 4 vs. 2.6%, n = 1; p = 0.36) in the unweighted analysis; three of the four cases in the distal DAC group were discovered immediately after thrombectomy. A patent artery, confirming there was no flow compromise, was observed on follow-up imaging; all patients had a good clinical outcome. However, the remaining case was one of fatal vascular injury, which resulted in death due to complications (Fig 3).

In that case, the DAC tip, although steam-shaped, was not able to pass through the origin of the ophthalmic artery. Moreover, the pushing forces required to navigate the DAC and aortic arch tortuosity resulted in herniation of the BGC. A stent-retriever was deployed over the thrombus for anchoring to advance the DAC. Additionally, the balloon of the BGC was inflated to provide an anchor and reduce catheter herniation. The DAC was subsequently pulled over the stent-retriever wire and advanced into the proximal MCA; however, the DAC could not be advanced any further. After the release of tension in the DAC, a thrombectomy was performed. The MCA was recanalized with mTICI = 3; however, a contrast extravasation was observed in the proximal MCA. In the proximal DAC group, arterial dissection was caused by wire manipulation during selection of the MCA. This patient did not suffer any serious complications associated with the dissection. Emboli in new territory were not observed in either groups.

On multivariate logistic regression analysis, the DAC tip position was independently associated with complete reperfusion (unweighted analysis: aOR, 4.10; 95% CI, 1.40–11.98; p = 0.01; weighted analysis: aOR, 5.20; 95% CI, 1.72–15.78; p = 0.004). Other parameters are summarized in Table 3.

**Table 3:** Multivariable logistic regression analysis with dependent variables for successful reperfusion, complete reperfusion in all cases (n = 81).

	Unweighted analysis				Inverse-probability-of-treatment-weighted analysis			
	Successful reperfusion (modified TICI≥2b)		Complete reperfusion (modified TICI = 3)		Successful reperfusion (modified TICI≥2b)		Complete reperfusion (modified TICI = 3)	
	Adjusted Odds Ratio (95% CI)	P value	Adjusted Odds Ratio (95% CI)	P value	Adjusted Odds Ratio (95% CI)	P value	Adjusted Odds Ratio (95% CI)	P value
Puncture-to-recanalization time (min)	0.981 (0.916–1.050)	0.573	1.032 (0.979–1.088)	0.243	0.967 (0.894–1.044)	0.387	1.013 (0.955–1.074)	0.671
Number of passes	0.321 (0.149–0.690)	0.004	0.307(0.145–0.650)	0.002	0.269 (0.117–0.617)	0.002	0.274 (0.127–0.590)	< 0.001
Collaterals from Acom or Pcom artery	0.224 (0.059–0.858)	0.029	0.360(0.121–1.071)	0.066	0.133 (0.029–0.605)	0.009	0.324 (0.105–0.998)	0.05
Distal DAC position			4.102 (1.404–11.982)	0.010			5.202 (1.715–15.777)	0.004
Age			1.026 (0.977–1.078)	0.298			1.033 (0.981–1.089)	0.214
tPA			3.017 (0.986–9.228)	0.053			4.178 (1.336–13.064)	0.014

95% CI: 95% confidence interval; ACom, anterior communicating; PCom, posterior communicating; DAC: distal access catheter; tPA: tissue plasminogen activator.

In the subgroup analysis of the proximal DAC group using multivariate logistic regression analysis, collateral flow from the ACom or PCom artery was the only independent factor influencing complete reperfusion (unweighted analysis: aOR, 0.064; 95% CI, 0.007–0.594; p = 0.016; weighted analysis: aOR, 0.091; 95% CI, 0.012–0.681; p = 0.02) (Table 4).

**Table 4:** Multivariable logistic regression analysis with dependent variables for successful reperfusion and complete reperfusion in the proximal DAC group (n = 38).

	Unweighted analysis				Inverse-probability-of-treatment-weighted analysis			
	Successful reperfusion (modified TICI≥2b)		Complete reperfusion (modified TICI = 3)		Successful reperfusion (modified TICI≥2b)		Complete reperfusion (modified TICI = 3)	
	Adjusted Odds Ratio (95% CI)	P value	Adjusted Odds Ratio (95% CI)	P value	Adjusted Odds Ratio (95% CI)	P value	Adjusted Odds Ratio (95% CI)	P value
Puncture-to-recanalization time (min)	0.907 (0.810–1.016)	0.092	1.061 (0.971–1.160)	0.190	0.901 (0.802–1.013)	0.081	1.050 (0.965–1.141)	0.258
Number of passes	0.657 (0.218–1.977)	0.455	0.422 (0.139–1.281)	0.128	0.690 (0.237–2.013)	0.497	0.339 (0.110–1.050)	0.061
Collaterals from Acom or Pcom artery	0.151 (0.021–1.057)	0.057	0.064 (0.007–0.594)	0.016	0.164 (0.026–1.052)	0.057	0.091 (0.012–0.681)	0.020

DAC: distal access catheter; TICI: Thrombolysis in cerebral infarction; 95% CI: 95% confidence interval; ACom: anterior communicating; PCom: posterior communicating.

In contrast, in the distal DAC group, the collateral flow from the ACom or PCom artery did not significantly influence successful reperfusion (unweighted analysis: aOR, 0.622; 95% CI, 0.062–6.261; p = 0.687; weighted analysis: aOR, 0.548; 95% CI, 0.091−3.286; p = 0.511), or complete reperfusion (unweighted analysis: aOR, 1.077; 95% CI, 0.230−5.040; p = 0.925; weighted analysis: aOR, 0.943; 95% CI, 0.267−3.324; p = 0.927). The number of passes was the only independent factor influencing both successful reperfusion (unweighted analysis: aOR, 0.104; 95% CI, 0.021−0.524; p = 0.006; weighted analysis: aOR, 0.081; 95% CI, 0.206−0.316; p < 0.001) and complete reperfusion (unweighted analysis: aOR, 0.267; 95% CI, 0.106−0.674; p = 0.005; weighted analysis: aOR, 0.197; 95% CI, 0.094−0.410; p < 0.001) (Table 5).

**Table 5:** Multivariable logistic regression analysis with dependent variables for successful reperfusion and complete reperfusion in the distal DAC group (n = 45).

	Unweighted analysis				Inverse-probability-of-treatment-weighted analysis			
	Successful reperfusion (modified TICI≥2b)		Complete reperfusion (modified TICI = 3)		Successful reperfusion (modified TICI≥2b)		Complete reperfusion (modified TICI = 3)	
	Adjusted Odds Ratio (95% CI)	P value	Adjusted Odds Ratio (95% CI)	P value	Adjusted Odds Ratio (95% CI)	P value	Adjusted Odds Ratio (95% CI)	P value
Number of passes	0.104 (0.021–0.524)	0.006	0.267 (0.106–0.674)	0.005	0.081 (0.206–0.316)	<0.001	0.197 (0.094–0.410)	<0.001
Collaterals from Acom or Pcom artery	0.622(0.062–6.261)	0.687	1.077 (0.230–5.040)	0.925	0.548 (0.091–3.286)	0.511	0.943 (0.267–3.324)	0.927
Smoking	5.929 (0.427–82.381)	0.185			5.663 (0.643–49.861)	0.118	0.498 (0.147–1.693)	0.264
tPA			2.468 (0.487–12.506)	0.275			2.369 (0.671–8.371)	0.180
Initial NIHSS score			1.040 (0.877–1.233)	0.651			1.094 (0.952–1.256)	0.205

DAC: distal access catheter; TICI: Thrombolysis in cerebral infarction; 95% CI: 95% confidence interval; ACom: anterior communicating; PCom: posterior communicating; tPA: tissue plasminogen activator; NIHSS: National Institute of Health Stroke Scale.

## Discussion

**Fig 4:**
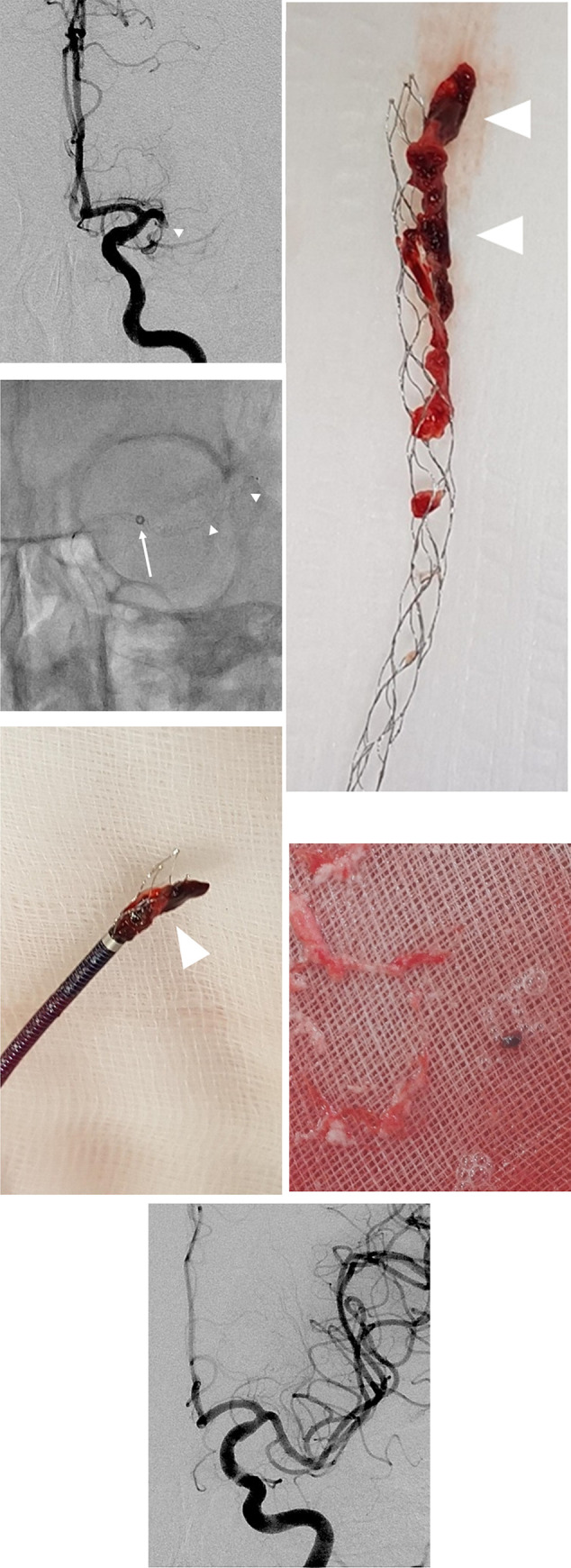
Combined stent-retriever and distal access catheter (DAC) technique. (A) Left anteroposterior internal carotid artery angiogram image shows occlusion of the left middle cerebral artery (arrowhead). (B) Mechanical thrombectomy was performed using stent-retriever and DAC. The DAC was advanced in the middle cerebral artery (arrow) and the stent-retriever (arrowheads) was deployed through the thrombus. (C, D) Thrombus within the stent-retriever (arrowheads) is partially captured within the DAC (E) Debris and clot fragmentation aspirated through the DAC (F) Post-thrombectomy anteroposterior internal carotid artery angiogram shows complete reperfusion of the middle cerebral artery.

Although there are two main methods for mechanical thrombectomy (stent-retriever and aspiration), there are several different techniques for the procedure. The mechanical thrombectomy techniques have continued to evolve for improvement in flow-control and aspiration force. Recently, the stent-retriever method, in conjunction with new approaches using DAC (Fig 4), has been used for better clinical and radiological outcomes in acute ischemic stroke [16–20]. The single-arm studies on the SAVE and ARTS technique have demonstrated a good rate of complete reperfusion (mTICI = 3; 56% and 54.8%, respectively) [16, 17]. In a recent study by Alex *et al*., a combined technique with SAVE was observed to be superior to ADAPT in achieving successful reperfusion (mTICI ≥ 2b in 93.5% vs. 75.0% cases) and complete reperfusion (mTICI = 3 in 28.3% vs 22.2% cases) [18]. With regard to the additional use of a BGC, in the studies on PROTECT and PROTECT plus, successful reperfusion was accomplished in all cases, with a complete reperfusion rate of 49.5% and 73.5%, respectively [19, 20].

The impaction force on the thrombus during thrombectomy is the primary factor determining the amount of force required to remove the clot. It is established by the pressure gradient across the clot, i.e., the systemic pressure minus the pressure from retrograde collateral blood flow at the distal thrombus face. The combined stent-retrieval and DAC technique can reduce the impaction force on the proximal surface of the thrombus, with the percentage of the parent artery lumen cross-sectional area occupied by the catheter before suction and with aspiration [21]. Thus, theoretically, aspiration with continuous negative pressure through a DAC catheter, during thrombectomy, may have a synergistic effect on successful reperfusion rates and lower distal thrombus migration. As reported in an *in vitro* study [22], the aspiration force increases upon contact of the DAC tip with the thrombus. In this study, the distal DAC group showed a better reperfusion rate than the proximal DAC group, which was theoretically expected in both the unweighted and weighted analysis. Moreover, the clinical outcome and early neurological changes were also better in the distal DAC group. DAC tip position was independently associated with complete reperfusion in both the unweighted and weighted analysis.

Pressure exerted on the proximal surface due to collateral flow via the ACom or PCom artery can also increase the pressure gradient across the clot [23]. According to an *in vitro* study, aspiration through a DAC or distal aspiration catheter, with balloon protection, is likely to reverse blood flow in the ICA and MCA [24]. Here, in all cases, the BGC was used to reduce the impaction force on the thrombus. However, in the presence of collaterals from the ACom or PCom artery, complete flow-arrest is usually not possible. In our study, in the proximal DAC group, the DAC tip was placed in the cavernous ICA below the ophthalmic artery origin. The collateral flow from the ACom or PCom artery in the proximal DAC group had a significantly negative impact on the complete reperfusion rate. The relative long distance between the clot and the DAC tip, and collateral flow from the ACom or PCom artery (if any) may have diminished the effectiveness of the DAC aspiration force. Thus, the DAC should be placed as close as possible to the proximal surface of the clot.

Various methods have been suggested to advance the DAC to the target occlusion in cases with tortuous vascular anatomy. One study suggested a technique that uses compliant balloons in a coaxial system with a large-bore aspiration catheter for easy navigation by reducing the inter-catheter space at the tortuous curvature of the artery [25]; another suggested an anchoring technique, where the stent-retriever is deployed over the clot, and the aspiration catheter is pulled over the stent-retriever wire [26]. Despite various methods, advancing the DAC through tortuous vessels may be challenging, and with excess manipulation, there is a potential risk of damage to the vasculature. In fact, we experienced four cases of vascular injury in the distal DAC group, where one patient died of complications. In this study, strong inadvertent pushing force during the advancement of the DAC, or the relatively large bore 6Fr catheter, may have contributed to the endothelial damage. Contrary to the distal DAC group, there was only one dissection case in the proximal DAC group that was not related to the DAC tip. Emboli in new territory were not observed in any of the cases. Moreover, in comparison with the SAVE study [18] for MCA occlusion, the complete reperfusion rate in the proximal DAC group was higher in our study (29.2% vs. 42.1%). Further, the benefits of the DAC have to be considered with the potential risks. Thus, if advancing the DAC close to the target lesion in the MCA is difficult, placing the DAC in the cavernous ICA, without worrying about complications, is worth considering.

The first-pass effect has been reported as an independent predictor of good clinical outcome after mechanical thrombectomy [7, 27]. However, our study demonstrated a numerically higher mean number of passes with good outcomes in the distal DAC group, although the finding was not statistically significant. This discrepancy may be accounted for in part by the higher complete reperfusion rate. Recent studies have suggested that successful reperfusion, rather than the number of passes, may predict clinical outcome after mechanical thrombectomy and should be the primary objective of stroke thrombectomy, irrespective of the number of passes [28–30].

This study has several limitations. First, it was a single-center study, where patients treated with one type of stent-retriever and DAC, to minimize the effects influenced by the type of devices, were enrolled. Other stent-retrievers and catheters may have varying degrees of diameter and softness, which may affect the risk of vessel injury. Further prospective studies, using variable devices, are warranted to demonstrate the efficacy and safety of the combined stent-retrieval and DAC technique. Second, several studies have demonstrated a correlation between the angiographic reperfusion rate and clinical outcomes [31, 32]. Here, a trend for better clinical outcome was observed in the distal DAC group, although its statistical significance could not be proven. Further studies with a larger cohort are warranted. Third, conventional angiography is considered the gold standard to evaluate collaterals. Here, the presence of collateral flow from the ACom or PCom artery was presumed based on the visibility of the contralateral A1 segment or ipsilateral PCom artery on angiography. Multi-vessel angiography with compression test was not performed to evaluate the collateral flow due to the longer procedural time. Although the ACom or PCom artery may be visible only on the compression test in some cases, we considered that the effect on the DAC aspiration force would be insignificant in those cases.

## Conclusion

Our study demonstrated that a more distal placement of the DAC results in better successful reperfusion (mTICI ≥ 2b) and a significantly greater complete reperfusion (mTICI = 3). The distal DAC group also had better clinical outcomes. However, more events of vascular injury were observed in the distal group.Thus, when using the combined technique for thrombectomy, the DAC should be placed as close to the target lesion as possible; however, if this is too difficult, then placing the DAC in the cavernous ICA may be a viable alternative.
